# How to Use the Six-Step Digital Ethnography Framework to Develop Buyer Personas: The Case of Fan Fit

**DOI:** 10.2196/41489

**Published:** 2022-11-25

**Authors:** Alex Fenton, Aleksej Heinze, McVal Osborne, Wasim Ahmed

**Affiliations:** 1 Business School University of Chester Chester United Kingdom; 2 Marketing Department KEDGE Business School Marseille France; 3 Flight Story London United Kingdom; 4 Management School University of Stirling Stirling United Kingdom

**Keywords:** health tracking, digital, ethnography, apps, mobile app, customer, physical activity

## Abstract

**Background:**

One of the key features of digital marketing is customer centricity, which can be applied to the domain of health. This is expressed through the ability to target specific customer segments with relevant content using appropriate channels and having data to track and understand each interaction. In order to do this, marketers create buyer personas based on a wide spectrum of quantitative and qualitative data. Digital ethnography is another established method for studying web-based communities. However, for practitioners, the complexity, rigor, and time associated with ethnographical work are sometimes out of reach.

**Objective:**

This paper responds to the gaps in the practically focused method of using social media for digital ethnography to develop buyer personas. This paper aims to demonstrate how digital ethnography can be used as a way to create and refine buyer personas.

**Methods:**

Using a case study of the Fan Fit smartphone app, which aimed to increase physical activity, a digital ethnography was applied to create a better understanding of customers and to create and refine buyer personas.

**Results:**

We propose two buyer personas, and we develop a 6-step digital ethnography framework designed for the development of buyer personas.

**Conclusions:**

The key contribution of this work is the proposal of a 6-step digital ethnography framework designed for the development of buyer personas. We highlight that the 6-step digital ethnography could be a robust tool for practitioners and academicians to analyze digital communications for the process of creating and updating data-driven buyer personas to create deeper insights into digital and health marketing efforts.

## Introduction

Digital communication channels offer great benefits to marketers for understanding customer behavior to bring customer centricity to the core of digital marketing strategies. The buyer persona concept was designed specifically for digital marketing strategies and is linked with the concept of the buyer persona spring, as outlined by Heinze et al [1]. Buyer personas can also be applied within the health domain and are useful for marketing health-related initiatives. The focus of this case study is in the health domain, which is increasingly becoming an important research area [2], more specifically in the segment of digital fitness and well-being devices, which is projected to reach over US $58 billion in 2022 and US $99 billion by 2027 [3].

Heinze et al [1] present the buyer persona concept as a fictitious representation of the customer segment, which has a number of elements allowing marketers to understand customer needs and how they would like to be interacted with. The core of the buyer persona insights is the pain points—the main issues that the buyer persona faces and attempts to alleviate through the use of the products or services of a particular organization. For each pain point, a corresponding trust point is also established, which helps to understand the perceived solutions to these problems.

The reasons why these solutions are *perceived* and not necessarily the definitive correct solutions is because buyer personas are based on aggregate knowledge from multiple sources, and buyers might be misinformed or simply unaware of the “correct” solution that is currently available on the market. Realizing these points also helps marketers to identify keyword phrases associated with these areas of problems and solutions and, as a consequence, estimate the size of the audience and the seasonality of these terms.

While the buyer persona concept is driven by practitioners, it is not discussed in the academic literature—only textbooks [4] that focus on how to use buyer personas. Other studies [5] have limitations in that they only use a simplistic approach and state that certain questions in relation to a buyer persona have to be answered without going in-depth on the process of answering these questions. A more comprehensive look at the buyer persona development was given by Revella [6], who acknowledges the need for multiple data sources, including interviews, customer surveys, and big data, in particular the use of loyalty cards and web analytics. While it is a comprehensive book that outlines buyer persona understanding and development, it misses two key data sources available to digital marketers—past keyword phrases searched and a methodical approach to studying social media.

Formulating and refining personas based on an ever-increasing volume of social media channels and content is an ongoing challenge for digital marketers because the digital landscape changes constantly. This calls for a flexible method that can adapt to understand customers now and in the coming years. This knowledge allows marketers to refine products and services and keep customer centricity at the center of digital and social media marketing activity.

Two key sources of data for buyer persona development are keyword research based on search engine use and social media analysis based on publicly available posts and engagement data. The process of refining and developing insights from search engine use data benefits from search engine keyword research tools such as Google Ads and the Baidu Index. Despite an established set of tools, there is still a need for interpretation of those search terms. The selection of buyer persona strategic terms does still require human analysis.

However, marketing automation tools that manage paid advertising campaigns do exist, and there are various options for larger-scale campaigns. The keyword research data is relatively well structured, showing when, where, and how often a particular search term was used when compared to social media. The use of social media targeting is more unstructured, on the other hand. No one particular method is apparent, but methods such as netnography [7] and digital ethnography [8] are well-established ways of understanding more about people and social media communities.

While these methods are well established for academic and practitioner research, they can be considered complex, time-consuming, and challenging to operationalize. Furthermore, the General Data Protection Regulation and ethical issues such as obtaining informed consent from each one of the observed individuals are complex and shifting landscapes. Netnography [2,7] provides the clearest and most established set of guidelines for conducting social media ethnographical research, and following these guidelines would enable buyer personas to be created and refined [1]. In this paper, however, we explore an alternative approach to digital ethnography. Ethnography as an approach is methodologically flexible; therefore, in this paper, we explore digital ethnography as a flexible approach to the practical creation of buyer personas.

A lot of brands might talk about “Net Zero,” but what does it mean in reality for a brand? There is a need to go beyond the numbers, starting with quantitative data but then flexibly returning to qualitative data and back again; this responsive adaptiveness is required to formulate and maintain buyer personas.

Therefore, this formative paper aims to propose and illustrate a 6-step digital ethnography (SSDE) as one of the methods for data analysis to create buyer personas. The paper is structured as follows. First, the concept of a buyer persona is reviewed and discussed. Second, the use of digital ethnography as a core method and its challenges are discussed based on the literature. Third, the application of digital ethnography is illustrated using the health case study of Fan Fit as an example.

## Buyer Personas

The traditional marketing literature has been concerned with the segmentation of the markets for decades. The idea that the same message cannot be shared with all prospective customers and customer segmentations is decades old [9]; in his article, Marcus [9] shares a method for dividing existing customers into groups based on past purchase frequency and discusses other statistical methods to divide customers into groups. While this method is useful for understanding customer behavior, it gives us a single dimension to understanding customers based on their past purchases. These kinds of segmentations suffer from the survivorship bias [10], where we only get to know the needs of those who are already our customers and not those who chose not to use our products and services. Further studies on customer segmentation tend to also focus on statistical analysis methods that allow for a more detailed understanding of usually existing customers [11]. Furthermore, these approaches are not suitable for small- and medium-sized organizations and start-ups that do not usually possess statistical know-how nor do they have large enough data sets of customers for querying. This is why the concept of a practical but methodical approach is necessary in digital marketing, and SSDE is therefore relevant and practical.

The buyer persona and buyer persona spring concept are outlined in the book *Digital and Social Media Marketing: A Results-Driven Approach* [1]. The buyer persona has much in common with other customer personas because it is a fictitious representation of a customer segment. Customers and potential customers come in various ages, races, classes, genders, and other demographics. Digital technologies and data capture are improving all the time in understanding and delivering personalized and timely information to people. Even so, marketers cannot understand everything about their audiences individually, so personas are of great value to marketers. While offering us a number of examples and suggestions on how to develop a buyer persona template, there is no methodical approach that helps in understanding the transparency of how these questions should be answered.

Buyer personas are similar to user personas, but the main difference is buyer personas are representative of the ones who will be “buying” into your ideas, products, or services as opposed to simply using something. For example, for children’s books, the user persona will consider how the child will use a book, whereas the buyer persona for the same child would focus on how to encourage the same child to use a book. The purchase process does not always need to be financial—it could be simply the “buying of a political idea” or understanding a concept. Buyer personas also state the role that this individual has in the purchase process—for example, a child could have a role of an influencer, while the parent has the role of a gatekeeper, decision maker, or purchaser. The buyer persona, therefore, is an invaluable way of better understanding audiences in order to create targeted products and service communications, and for health marketing purposes.

Two potential approaches for the creation of buyer personas are science fiction prototyping and data-driven development. Science fiction prototyping is usually used by organizations that want to become leaders in the market through disruptive innovation. These organizations often develop products or services for which there is no current customer segment, which makes creating a real-life buyer persona impossible. For example, when developing software products for the management of yet-to-be-invented robotics systems, AutoDesk used science fiction prototyping [12] in its buyer persona development. This is because there was not yet anything like this product on the market and, therefore, a lack of data as to what drives customer purchase decisions before the product even exists.

**Figure 1:**
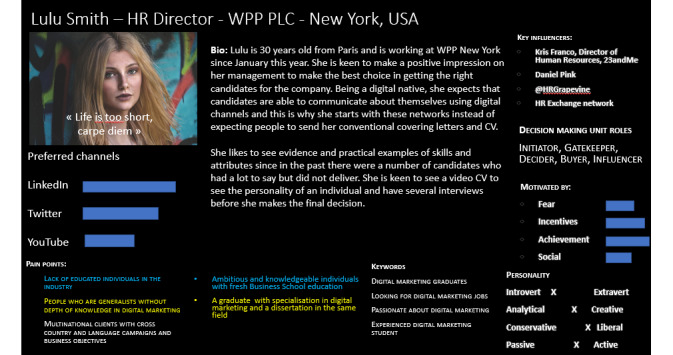
An example buyer persona.

Alternatively, the data-driven buyer persona is based in areas where an existing product or service does exist on the market. The content of a buyer persona generally includes demographics, average order value, locations, national and regional culture, socioeconomic background, and decision-making patterns. Central to buyer persona information are the “pain touch points” and corresponding “trust touch points.” It should also include the keywords they might use on search engines, the social networks that they might engage with, and other information that helps marketers to focus content based on buyers’ needs. The ability to target and select specific and unique audience segments is being made possible through the proliferation of digital communications and social media channels. The purpose of developing a detailed buyer persona is, therefore, to better know this target audience through this technique of digital personification. Figure 1 provides an example of a buyer persona.

There are several ways to develop a buyer persona using a data-driven strategy. The data-driven process uses real data to understand the pain points and corresponding hot points or trust points of the buyer persona. Data from surveys and publicly available data can be used to develop the profile. Social media is also a critical source in order to understand the customer. For the social media marketer, interacting with customers is generally a very regular occurrence, and this participation is critical to developing buyer personas. A key aspect of ethnography is participant observation and interaction with people. Digital ethnography is therefore particularly suited to engaging with an audience and for observing, understanding, formulating, and refining buyer personas.

## Digital Ethnography

Ethnography as a research method has a long history in studying people and storytelling [8]. Some consider it the most in-depth of all possible research methods because it “enables a researcher to see what people are doing as well as what they say they are doing” [8]. Digital ethnography is a newer branch of ethnography that takes into account digital communities as a key focus [4]. Various forms of ethnography are dedicated to studying technology and digitally enabled communities. Digital ethnography was selected for this paper because it has long been established [6] as well as its focus on “yielding new insights for our understanding of data” [4] and its practical flexibility. The *Routledge Handbook of Digital Ethnography* [4] is a useful guide to applying this method and states that “Drawing from the empirical, ethnography can provide insight into motivations and practices that in turn shape future directions for digital media.”

The application of digital ethnography to create buyer personas shapes the direction of digital marketing practice and campaigns. Therefore, it is an important concept for developing and refining buyer personas. This is critical to the formulation and execution of digital marketing strategies.

Table 1 outlines the 6 steps of digital ethnography used over the past 5 years, from 2017 to 2022, to teach digital marketing students in higher education institutions in the United Kingdom and France.

This teaching was done at specialized master’s programs with digital marketing courses as well as for MBA students. About 300 students per year have taken these courses at internationally recognized business schools. Students were tasked to create a buyer persona for a particular case study, whereby the group was recommended to focus on different buyer personas for the same brand. This allowed participants to undertake the same process for a range of different markets—both business-to-business as well as business-to-consumer. One of these live case studies was the Fan Fit case, where students had the option to expand the application of the market currently relevant to the Fan Fit white-label app. The steps and tasks for students were refined over the years to facilitate a clearer process that works for all cases.

The table was cross-referenced with practices used by one of the largest digital marketing agencies that used buyer personas on a regular basis for all of their communications.

**Table 1:** Six-step digital ethnography framework.

Stage	Tools and techniques	Outputs for buyer persona
(1) Ideation: documenting views of the potential buyer persona	Brainstorming of buyer persona elements—there are no data, just ideas of individuals who undertake this work	All elements of buyer persona based on the perceptions and preconceptions of the initial idea
(2) Social proofing: first impressions of real data existence for relevant topics	Initial observations of potential online communities and reviews of products or services; hashtag analysis, online reviews for related products and services among your own and competitor communications such as social media channels	Confirmation, disproof, and identification of new themes for pain and trust points; pictures and quotes of real issues expressed online by potential buyer persona representatives
(3) Horizon scanning: industry reports, analysis, and wider statistics	Reports done by market analysis firms such as Mintel and MarketLine, and wider statistics such as those offered by governments or panel data such as Statista	Confirmation, disproof, and identification of new themes for pain and trust points, adding more contextual data through wider reports and statistical publications, if available
(4) Keyword research: using search data to see evidence	Using search engine past search behavior trends as an indication and prediction of interest; keyword research tools such as Google Trends and Google Keyword Planner or Baidu Index for the Chinese market	Identification of keywords with potential demand, which could be integrated into the content development for the buyer persona; understanding their terms’ use and potential seasonality
(5) Content audit: skyscraper content and influencer identification	Using identified keyword terms confirming most visible content, places, and individuals/organizations who are engaging existing buyer personas; various tools can be used in this stage	List of channels, content examples (screenshots, links, and evidence of its popularity); list of influencers who regularly engage on these topics using identified keywords
(6) Proofing: finding evidence that the updated buyer persona exists	Using focus groups, surveys, and social concept tests to identify if the issues highlighted in the buyer persona research reflect the reality of the target audience	Confirmation and update of the buyer persona understanding; refinement of the buyer persona as necessary

## Fan Fit Health Case Study

**Figure 2:**
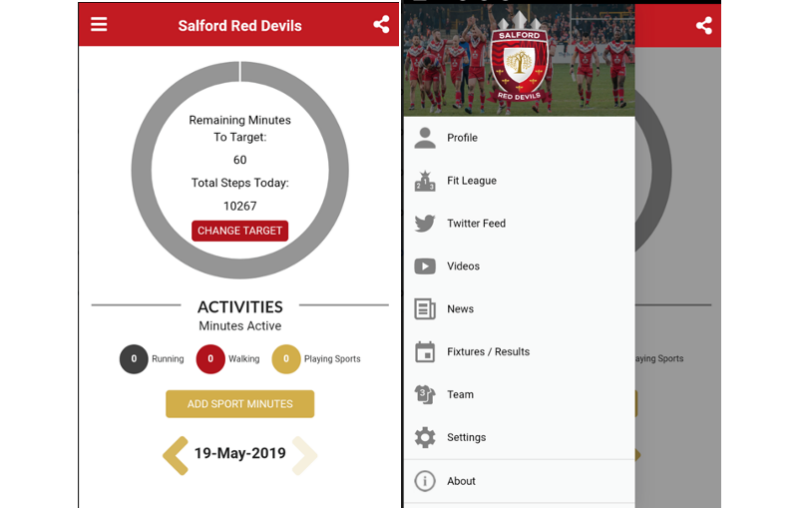
Fan Fit Red Devils smartphone app.

The main case study used in this section is “Fan Fit” [13]. This case study is used as an example, and an overview of the app is shown in Figure 2.

Fan Fit began in 2015 as a project started at the University of Salford in the United Kingdom, which aimed to use technologies to engage fans and customers around fitness and well-being. The first major implementation of the project was a smartphone app created for Android and iPhone platforms, which eventually became the first and only official club app of the Salford Red Devils Rugby Club. Red Devils are a top-division Super League club that, like many sports clubs, had a website and social media channels but no smartphone app to engage with their fans.

The app offered the usual sports club app functionality of pulling in news, fixtures, and social media. A key differentiating feature between this and other similar apps was the addition of an activity tracker for measuring walking and running automatically through the phone or a wristband. This enabled the app to create fitness leagues between fans (global, monthly, or personal), gamifying user engagement. Using gamification principles, fans could win digital badges and physical prizes such as season tickets, shirts, or fitness wristbands through the app, creating a social media buzz and health-focused dialog between the club and fans. The project used a human-centered design approach, involving fans at each stage of development through surveys, focus groups, and online and offline participant observation.

Ultimately, the project aimed to:

- engage fans through interactive technology to build a new kind of brand community and capture new types of user data, and
- raise awareness around major public health challenges related to heart disease and obesity while encouraging fans to be more active and develop healthier lifestyles.

To develop a minimum viable product that sports clubs could test, the Fan Fit team integrated fitness-tracking technology into a brandable (white labeled) official club app. This allowed them to obtain a “first mover advantage” and bring a new product to market before other competitors could do the same. This minimum viable product opened up doors to other opportunities—discussions with sports clubs and the first major sign-up with the Salford Red Devils. This success created opportunities at the national level to get further funding and the signing of a major football club (Rangers FC).

A key aspect of the Fan Fit project was the creation of a digital marketing strategy for the project to reach new sports clubs and other organizations interested in the project (business-to-business). In addition, for the version of the app that existed, a business-to-consumer digital marketing strategy was used to reach fans of those clubs.

In all cases, the research team used a combination of surveys, focus groups, and ongoing digital ethnography to understand more about the audience and formulate and refine buyer personas. Having outlined the importance of the buyer persona, we now illustrate how this can be applied to our case of Fan Fit. Two buyer persona examples are shown, one for the fan (business-to-consumer) and another for a sports club (business-to-business).

## Buyer Persona for Fan Fit: Jim Smith (Fan)

Jim Watson is a 52-year-old man from Salford. He is a season ticket holder with the Salford Red Devils and attends most home games. He is also an avid football fan, attending some Manchester United matches and following them on TV. He also attends some matches of the Salford FC. Jim is a van driver in Salford, his wife is Marie, and he has three grown-up children and two grandchildren. 

### Channels

Jim uses television, Twitter, Facebook, and YouTube to find the latest news on his favorite teams, keep in touch with family, and interact with other fans on Facebook and Twitter.

### Frustrations and Pain Points

Jim has thought about buying a fitness wristband and is trying to be more active and develop healthier habits, but he does not know which one to buy or how to set up or use it. His son has a FitBit but is always very busy with his work and children, so Jim does not want to bother his son by asking for a recommendation. 

### Hot or Trust Points

Jim has become more aware of the importance of fitness after his friend had a minor heart attack last year. He thinks a mobile exercise tracking app can help him prevent ill health and improve his quality of life.

## Buyer Persona for Fan Fit: Andrea Rogers (Club)

**Figure 3:**
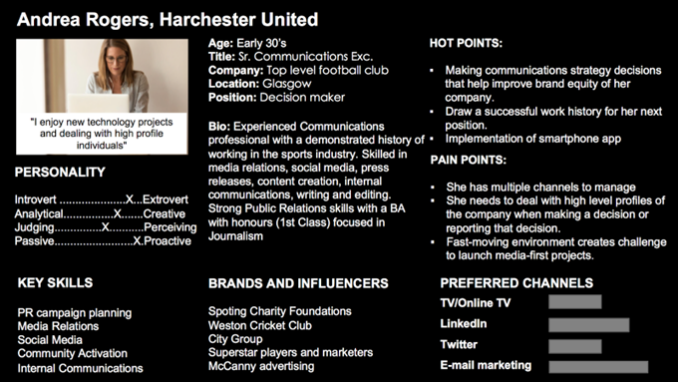
Buyer persona for Andrea Rogers.

Andrea is a 33-year-old woman originally from Manchester (Figure 3). She is a senior marketing and communications professional with a major football club. She manages a small team who runs the club website and social media channels, and a wider pool of journalists and content producers. She is recently married but does not have any children.

### Channels

Andrea uses television, Twitter, Facebook, YouTube, Instagram, and LinkedIn. She uses Facebook and Instagram primarily to keep in touch with her friends and family. She uses Twitter and LinkedIn primarily as her professional network and to seek new opportunities and promote initiatives.

### Frustrations and Pain Points

Andrea is acutely aware that her club has no plans for a smartphone app and has started to fall behind other rival clubs with their use of innovative technologies and digital fan engagement.

### Hot and Trust Points

Andrea is interested in new technologies such as apps, the Internet of Things, virtual reality, augmented reality, eSports, and anything that would potentially bring the club to a new audience of young fans. She would also like to engage more female fans through digital channels, as they are not proportionately represented on social media. 

## How These Buyer Personas Were Developed: Data

Digital and social media channels like Google, Twitter, LinkedIn, Instagram, Facebook, and Reddit provide marketers and health researchers with treasure troves of information on their target audiences. The content published across these channels can be exhaustive in terms of scope and detail, and the data captured from this content can be incredibly valuable in producing accurate buyer personas. Data come in all kinds of shapes and forms. In terms of the buyer persona spring and as part of the digital entrepreneurship strategy, we are most interested in the data sources that help to better understand the buyer persona, the channels, and the content. Data sources, including things like websites and social media analytics, can be invaluable sources of information and insight. Data in the raw format must be cleaned and visualized in such a way that they can be used to add meaning to the strategy in an ongoing way. 

Data-gathering strategies can also be devised to create new primary data sources. For example, making a survey whose results can be visualized is a simple way to gather some useful quantitative data to inform the strategy and answer many questions; in order to delve deeper and answer questions relating to your digital strategy, it may be necessary to also gather some qualitative data derived from your audience, experts, or the general public. This kind of qualitative data can be derived from interviews, focus groups, and even through social media analysis of comments and posts, including netnography and digital ethnography.

The ultimate goal of a data monitoring strategy is to keep track of achieving strategic business objectives. Strategic in this sense means that they are long-term—perhaps 1 year, 3 years, or 5 years long. The longer the time span, the more it is necessary to have key performance indicators along the way to see if these objectives are going to be reached. Using the SMART objective setting helps to make them more transparent.

## Conclusions

Fan Fit, our health-based case study, has at least two distinct buyer personas. The first one is fans (eg, Jim Smith), and the second is marketing and communications staff and decision makers within organizations that may want to adopt a version of Fan Fit for themselves (Andrea Rogers).

In the buyer personas outlined above, we demonstrated which channels Jim and Andrea are using. In the case of Fan Fit, for example, Twitter may be an appropriate channel to reach Jim. In terms of content, combining official messages from the brand he follows (Red Devils) and the conversational approach among fans, Fan Fit founders, and the club would help build the social capital and community to reach Jim.

A competition to win a season ticket and the competition with his friends and fellow fans are enough to get him to download the Fan Fit Red Devils app and get more active and engaged. In Andrea’s case, LinkedIn may be an appropriate channel to identify who she is and send her a personal connection request and a personalized message about Fan Fit. It is also possible to produce press releases and share these more widely using LinkedIn. Our findings are likely to be of interest to health marketing researchers and practitioners looking to promote positive health behaviors.

This paper has outlined a key methodological area that allows students, practitioners, and researchers to follow an approach to finding the answers to the questions around buyer persona development. The challenges highlighted in previous studies (eg, [9]) are reduced in the current process in that we are exploring data sets that are mainly openly available. The previous studies had survival bias, where classic customer segmentation approaches use the statistics of only existing customers. By reaching out to multiple data sources and cross-referencing these, we are supporting the ideas advocated by Revella [6] and developing a combination of data sources in a more digital ethnography-focused way.

The limitation of this study is that the method originated from discussions with teachers looking for a systemic, practical, and effective way to help students develop digital marketing strategies. The traditional methods of segmentations and buyer persona development were not accessible to learners as well as smaller organizations that do not have large internal databases to interrogate and develop their buyer personas. The practitioner perspectives are also only taken into account by one organization that has offices in Germany, the United Kingdom, and the United States. Other geographic areas, including the growing African digital ecosystems and Chinese perspectives, can add further richness to the application and further generalization of the framework.

Researchers are invited to explore and test the use of the SSDE in other settings. Practitioners can use the 6 steps of ideation, social proofing, horizon scanning, keyword research, content audit, and proofing for structuring their activities and offer transparency to the way in which their buyer personas are presented.

## Abbreviations

SSDE: six-step digital ethnography

## References

[1] Heinze A, Fletcher G, Rashid T, Cruz A (2020). Digital and Social Media Marketing: A Results-Driven Approach.

[2] Quévat A, Heinze A (2020). The digital transformation of preventive telemedicine in France based on the use of connected wearable devices. Glob Bus Org Exc.

[3] (2022). Statista.

[4] Cruz A, Karatzas S, Heinze A, Fletcher G, Rashid T, Cruz A (2020). Understanding your buyer persona. Digital and Social Media Marketing: A Results-Driven Approach.

[5] Akre V, Rajan A, Ahamed J, Al Amri A, Al Daisi S (2019). Smart Digital Marketing of Financial Services to Millennial Generation using emerging technological tools and buyer persona.

[6] Revella A (2015). Buyer Personas: How to Gain Insight Into Your Customer’s Expectations, Align Your Marketing Strategies, and Win More Business.

[7] Kozinets R (2019). Netnography: The Essential Guide to Qualitative Social Media Research.

[8] Hjorth L, Horst H, Galloway A, Bell G (2017). The Routledge Companion to Digital Ethnography.

[9] Marcus C (1998). A practical yet meaningful approach to customer segmentation. J Consum Market.

[10] Garcia C, Gould F (1993). Survivorship bias. J Portfolio Manag.

[11] Cooil B, Aksoy L, Keiningham TL (2008). Approaches to customer segmentation. J Relatsh Mark.

[12] Atherton E, Johnson BD (2016). Science fiction prototyping at work. Computer.

[13] Fenton A, Cooper-Ryan Am, Hardey Mm, Ahmed W (2022). Football fandom as a platform for digital health promotion and behaviour change: a mobile app case study. Int J Environ Res Public Health Interne.

